# Winnipeg-based elementary school teachers’ perspectives on food allergy management: a qualitative analysis

**DOI:** 10.1186/s13223-023-00798-z

**Published:** 2023-07-14

**Authors:** Mae Jhelene L. Santos, Kaitlyn Merrill, Natalie Riediger, Elissa M. Abrams, Nathalie Piquemal, Elinor Simons, Jennifer L.P. Protudjer

**Affiliations:** 1 Department of Food and Human Nutritional Sciences, University of Manitoba, Winnipeg, Canada; 2 Children’s Hospital Research Institute of Manitoba, Winnipeg, Canada; 3 Department of Pediatrics and Child Health, Rady Faculty of Health Sciences, University of Manitoba, Winnipeg, Canada; 4 Section of Allergy, Department of Pediatrics & Child Health, University of Manitoba, Winnipeg, Canada; 5 Faculty of Education, University of Manitoba, Winnipeg, Canada; 6 George and Fay Yee Centre for Healthcare Innovation, 501G-715 McDermot Avenue, Winnipeg, MB Canada; 7 Institute of Environmental Medicine, Karolinska Institutet, Stockholm, Sweden

**Keywords:** Anaphylaxis, Elementary school, Epinephrine, Food allergy, Food allergy management, Interviews, Qualitative study, Teachers

## Abstract

**Background:**

Food allergy affects 7-8% of children worldwide. Teachers supervise children in school, where most children spend their day. Yet, teachers have variable food allergy-related knowledge.

**Objective:**

We aimed to identify how Winnipeg-based elementary school teachers manage food allergy and prevent food-triggered allergic reactions in their classrooms and schools.

**Methods:**

Kindergarten-Grade 6 public and private school teachers, from Winnipeg, Canada, were interviewed virtually upon providing written informed consent. Interviews were recorded and transcribed verbatim. The study followed a pragmatic framework. Data were analysed via thematic analysis by multiple researchers.

**Results:**

We interviewed 16 teachers, who primarily identified as female (87.5%). Most teachers worked in public schools (87.5%) and, on average, had 5.8 years of teaching experience. We identified four themes within the data. Most teachers (68.9%) had direct or indirect experience with food allergy. Theme 1 described the minimal standardization and inconsistent enforcement of food allergy policies between and within schools. Teachers also had varied food allergy knowledge. Theme 2 reflected teachers’ variable confidence/perceived knowledge towards food allergy management, including feeling of stress and anxiety. Theme 3 captured the lack of standardized food allergy education for teachers, and concerns about the adequacy of the current provincial program. Theme 4 described how teachers spoke of relying on other school staff, families and students to have effective communication.

**Conclusion:**

Teachers’ food allergy management was informed by their knowledge and lived experience, guided by their school policies and individualized students’ needs. Teachers identified gaps in knowledge and communication, and desired more training and resources.

**Supplementary Information:**

The online version contains supplementary material available at 10.1186/s13223-023-00798-z.

## Introduction

Food allergy, a potentially fatal adverse food-induced immunological reaction [1], is a global public health concern. Food allergy affects an estimated 7.0–8.0% of children [2, 3], most of whom are diagnosed in early life. This prevalence translates to about 1–2 students per average-sized Canadian classroom [4]. In Canada, recent physician-diagnosed and/or history-based food allergy prevalence estimates are comparable to those of other countries. Notably, there has been increased self-reported or parent-reported food allergy [3, 5], which may be attributed to parents’ heightened awareness of food allergic symptoms (or gastrointestinal conditions that may present similarly), descriptions of food allergic symptoms in the media, and, longer wait times for diagnosis and adoption of early introduction to foods [5, 6]. Additionally, school age children (> 5 years) may be allergic to new foods as food exposure generally increases as the child gets older. To date, prevalence studies remain ambiguous in study design, methodology, populations and focus on different foods, presentation of symptoms, and factors influenced by geographic differences [6], race and socio-economic status [2].

Food allergic reactions, including anaphylaxis, may be life-threatening. Anaphylaxis is a severe systemic reaction that involves multiple organ systems [7]. In the general North American population, estimates of anaphylaxis vary around 0.05% [8] to 5.1% [9]. Pre-Coronavirus disease 2019 (COVID-19) pandemic, approximately 20.0% of reported anaphylaxis reactions amongst children occurred in school settings, where children spend most of their waking hours [10, 11]. Waserman et al. (2021) estimated that schools (average of 350 students) may have a median of 1.3 allergic reactions per school each year [12]. Therefore, it is crucial to investigate if and how schools support teachers and provide safe spaces for children with food allergy.

We previously reported teachers and school staff’s varied knowledge, experience and confidence levels in managing food allergy in their classrooms and schools [13]. The use of Emergency Anaphylaxis Plans (EAP), and availability and administration of epinephrine auto-injectors (EAI) during anaphylactic emergencies were also reported as underutilized in schools [13, 14]. Regrettably, fatal anaphylaxis has been reported in schools [15, 16], which highlights the need, and urgency, of critically evaluating how teachers, as the primary adults caring for children in schools, manage food allergy.

Further, laws and policies on food allergy management vary amongst Canadian jurisdictions (e.g. Ontario and Alberta) [17, 18]. Winnipeg, Manitoba, where this study was conducted, has no provincial, or city-wide policies on food allergy management, although the provincial Unified Referral and Intake System (URIS) program provides annual food allergy and anaphylaxis treatment training for employed teachers. Furthermore, food allergy policies may also exist within schools and public school divisions.

Teachers’ experiences and perception of food allergy management may be a complex phenomena, shaped by one’s personal experiences, coupled by diverse knowledge and management policies related to food allergy. Yet, there are limited qualitative studies available on teachers. Qualitative methods are necessary to investigate teachers’ experiences and to examine unique occurrences specific to food allergy management. Thus, we sought to answer how Winnipeg-based elementary school teachers manage food allergy and prevent food allergic reactions, including anaphylaxis, in their classrooms and schools.

## Methods

### Study design and population

As part of a larger project that aimed to describe the mental health impact and needs of children living with food allergy, and their caregivers, we interviewed elementary school teachers from Winnipeg, Manitoba, Canada using qualitative methods. This design allowed for a deeper understanding of teachers’ experiences and perceptions managing food allergy. The aim of qualitative methods is to gain perspectives of a specific subject or population, yet is applicable to similar contexts and settings [19]. Additionally, conducting interviews permitted teachers’ stories to be heard and explain the gaps where changes in practice were deemed necessary [19].

Teachers who taught in a Winnipeg-based public or private elementary (Kindergarten [starts the year the child turns age 5 years] to Grade 6) school were eligible. For the purposes of our study, we restricted recruitment to teachers in elementary schools only, as students in middle and senior school (i.e. junior and senior high school) are often not supervised during lunchtimes to the same extent as younger children. Moreover, students in elementary school typically have the same teacher for the majority of their classes, creating a situation where the teacher is highly likely to know each individual student and, where applicable, their food allergies. In contrast, students in middle and senior school commonly have different teachers for their classes, introducing the possibility that not all students with food allergies are known to teachers. Employed teachers on leave (i.e. parental leave) were also eligible. Teachers were recruited via social media and word-of-mouth between November 2021 and April 2022, in keeping with public health guidelines and closures [20] during the data collection period. The sample size was determined sufficient when data saturation has been achieved [19].

### Data collection

Potential participants were sent study information, screening eligibility questions and a consent form. Upon written informed consent, a mutually-convenient interview time was established. The project lead and student researcher (JP and MS, respectively) conducted the interviews using Microsoft Teams. Interviews followed a semi-structured guide (see eSupplement 1). Interviews were audio recorded and transcribed verbatim. Participants were provided a $30 e-gift card.

The interview guide provided the opportunity to ask participants similar questions, as consistent as possible, but were relevant to the research question. Semi-structured interviewing is flexible; the participant can elaborate on topics that may be valuable to the participant [19] and the interviewer is able to ask follow-up questions to seek further clarification.

To consider differences between schools by socio-economic status, we used the proxy of public- vs. private schools. In Manitoba, all children have the right to publicly-funded education [21]. In contrast, private schools often carry an additional tuition fee, often partly supported by private funds.

### Theoretical framework and data analysis

The study followed a pragmatic framework, which allows the researcher to use data collection and analysis methods that best solves real-world problems [19, 22]. The researcher acknowledges that there are multiple realities based on socially constructed experiences and the researchers’ worldview can influence the project [19]. Pragmatism was the chosen framework as it suited the objectives of this study, including finding actionable ways to enhance food allergy management in school settings.

Data were analyzed via thematic analysis, an active and inductive method to identify themes across a dataset [23]. Thematic analysis by Braun & Clarke (2006) supported the pragmatic framework, in that this analysis method is flexible and accessible, yet rigorous and provides organization of complex datasets [23].

Coding was independently completed by two researchers (initials blinded for review) using a codebook that was developed and agreed upon by the research team. Themes were actively identified within the data, and were not emerging concepts [23]. When no new or additional constructs were identified with subsequent interviews, we determined that data saturation was reached at 16 participants.

Rigor was defined by ongoing peer debriefing, reflexivity and research triangulation amongst the two researchers. Member checking was conducted to confirm the research findings to enhance study credibility [24]. This study was approved by the University of Manitoba Health Research Ethics Board (HS22242 [H2018:405]).

## Results

### Participant characteristics

We interviewed 16 teachers (Table 1).

**Table 1:** Participant characteristics

		n	%
Sex	Male	2	12.5
	Female	14	87.5
Personal experience with food allergy*	Direct,	5	31.3
	Indirect	6	37.5
	None	5	31.3
School type	Private	2	12.5
	Public	14	87.5
Income level of school area^**^	Lower income	7	53.8
Grades taught***	Kindergarten - Grade 3	14	-
	Grade 4–6	5	-
Type of class	Same age	8	50.0
	Multi-age	8	50.0
Years of teaching experience	< 5 years	5	31.3
	> 5 years	8	50.0
	Not reported	3	18.7

*Direct personal experience refers to participants who reported they had or have a food allergy, while indirect personal experience refers to those who had partners, family members and/or friends who had or have a food allergy. Participants who did not explicitly disclose personal experience with food allergy were counted under the category “no experience”

**School income areas were compared based on the low income cut-offs, after tax [21] for N = 13 teachers who taught in public schools

***Does not total N = 16; some teachers taught in multiple classes and/or grades

Most teachers identified as female (87.5%), and taught Kindergarten-Grade 3. On average, teachers had 5.8 years of teaching experience. Most teachers (11/16; 68.9%) had direct (i.e. reported food allergy history) or indirect (i.e. family member or friend who had food allergy) experience with food allergy.

### Themes

We identified four themes within the data.

#### Theme 1: “each classroom is a case-by-case basis”

This theme describes how teachers spoke of little standardisation of food allergy-related policies between and within private and public schools, public schools within the same divisions and classrooms within the same school. This theme also captures teachers’ decision-making in enforcing, and adhering to existing food allergy policies (Table 2).

**Table 2:** Qualitative themes, summary statement, codes and supporting quotations related to Theme 1: Each classroom is a “case-by-case” basis

**Theme description:** This theme describes the minimal standardization and inconsistent enforcement of food allergy policies between school divisions, schools within the same division, and classrooms within same school. This theme also captures the individual decisions made by teachers (and administrators) to manage food allergy in their classrooms and schools, both to adhere to school and/or school division-enforced policies, and policies that teachers have enforced individually (i.e., peanut/ tree nut bans and adding additional bans depending on students’ allergies).	
**Codes**	**Supporting Quotes**
*Ways to manage food allergy in the classroom*	I’m always checking in on those kids, even if it’s something that I know they’ve eaten a hundred times without nuts. If it doesn’t come from their home, I’m constantly going to them… “How you feel? Feeling good? Do you need water? Oh, I noticed you coughed just then. Are you okay? Oh, you just swallowed the wrong way. I’m sorry I’ll leave you alone now.”… Sort of hyper focus on those kids. (T12)
*Mealtime at school*	So we used to eat in a large lunch room [pre-pandemic]. All of the grade 3/4/5 students would eat in one room. We just have a blanket policy for allergies; peanuts and nuts are always a no-go. But if we had a child with a seafood allergy or something, we just wouldn’t allow it in that lunchroom. (T7) The lunch program is a blanket no nuts policy […] if [lunch supervisors] find nuts in someone’s food, they will ask the student to eat in the hallway or in the office. (T12)
*Implementation of policies related to food allergy management*	There’s no discussion about [food allergy management] ‘cause everyone has a different opinion [laughs] It just changes every two seconds, to be honest with you. So I think we just make up our own lines. Some err on the side of caution, some are more like, “Okay well if [student] is not ingesting [allergen], they’re fine”. (T16) I’ve seen different environments where allergies are not as high of a concern, and then schools where the classroom rules are very stringent. (T9) In each classroom, [food restrictions] are a case-by-case basis. (T11)
*Special events*	I would give the student with the allergy something else [instead of classroom treat] so they’re not completely left out. But, again, I would have to I think use my judgement with the kind of food it was and if there’s no indication at all, about like, “may contain” then I maybe send the [treat] home with the kid who brought the [treat]. (T11) It definitely make me feel a lot more anxious when we’re having celebrations where food is involved [.] I always feel a sense of anxiety, and I’m always checking in on those kids, even if it’s something that I know they’ve eaten a hundred times without nuts. (T12) [On planning field trips and managing risk], it’s balancing how can I be proactive and try to determine where the highest risk might be, and also mitigate that, but also, not single out the child too much […] there’s always some level of risk […] and you know it’s not helpful to put them into a bubble and not let them experience life because of that. (T14)
*Responding to food allergy emergencies*	The [students with food allergy] both carry an [EAI] on their person so that’s obviously accessible […] I am trained on how to use the [epinephrine auto-injector], but I would probably be a little bit overwhelmed in the [emergency] situation. I would like somebody else who is also trained in it to make sure that I’m doing it correctly, or if I’m not able to, that they are able to do it. […] It’s just a lot to deal with that - in that situation. Like I don’t want the kid in that situation– it just makes me anxious to think about it but when the time comes, I might be completely fine or I might pass out [laughs]. (T11) I haven’t really had incidents happening. So you can go through the whole year and be like, “Oh yeah. That was great, I had my training. I was prepared if something happens. But nothing happens.” So maybe that’s why I felt fine. If something were to happen mid-year, would I still feel comfortable remember how to use an [auto-injector]? (T20) [A student was] having a pretty severe reaction, I would say, but still able to know what […] he needed to do. He was a little bit older. This was a grade three student. He had eaten something in the classroom. I guess um, it had come into contact with something he was allergic to. I believe it was peanuts. He was able to let me know that something was wrong, and we got his [auto-injector] as he was carrying it on him, in a little pouch, and he administered his medicine. And I took him to the office, and he stayed there for further care. […] Even if it’s scary, you kind of have to put that to the side for a second and just refocus, um, and then you can freak out later when everything’s okay. […] Sometimes you might be the only adult like, around, and um, it – that just undermines the importance of um, trying to keep yourself calm and not letting your –your emotions, or whatever it is - fear, or the stress of the situation take over. (T9)

Abbreviations: *EAI* = epinephrine auto-injector; *T* = teacher

Birthdays, field trips and special events required extensive planning and communicating with families, which sometimes caused anxiety. Teachers talked about not having *“a lot of supports for managing [food allergy emergency].” (T7)* In general, teachers addressed food allergy-related situations on “*a case-by-case basis.” (T11)*.

Teachers talked about having *“blanket policies” (T7)* for managing peanuts and tree nut allergies, compared to other allergens. Teachers with students with various food allergies implemented additional food bans for their classrooms. Teachers described how they assumed responsibility in communicating classroom-level food allergy policies with families. One teacher described the ensuing confusion when certain situations occur, such as when a student brings in an allergenic food, for which there was *“no discussion about that ‘cause everyone has a different opinion” and then*, “*deal with it when it gets there*.” *(T16)*.

Mealtime management also differed between public schools. Teachers watched students during snack time. At lunch, as teachers are also on break, lunch supervisors or educational assistants (EA) primarily supervised students. However, teachers expressed concerns about the limited adult-to-student ratio, which may promote food sharing.

Some public schools also participated in subsidized meal programs wherein students can access breakfast and/or snacks and donated lunches. Descriptions of mealtime supervision and food provision was likewise different among private school teachers. One private school’s cafeteria provided all foods, including snacks and special treats, for all students, although this is atypical.

#### Theme 2: food allergy-related knowledge, experience and supports shape teachers’ confidence

Teachers described various levels of their perceived confidence related to allergy emergency management strategies, which was influenced by teachers’ food allergy knowledge and personal experiences. Teachers also relied on their students’ age and the involvement of families and supports from school staff (Table 3).

**Table 3:** Qualitative themes, summary statement, codes and supporting quotations related to Theme 2: Food allergy-related knowledge, experience and supports shape teachers’ confidence

**Theme description:** This theme encompasses the variable confidence/perceived knowledge of teachers towards allergy management, particularly emergency management strategies. Teachers’ confidence is largely based on personal experience with allergy (direct or indirect) and by supports available from the school (i.e., other staff involvement), family involvement, communication and personal attitudes, beliefs and experiences related to food allergy.	
**Codes**	**Supporting Quotes**
*Family involvement*	I always leaned on the parents with the kids with allergies and I always have them be my expert panel […] If I ever needed something I’d say, “Hey what’s your recommendation for this?”, “How do you think I should handle that?” (T17) I think that’s what’s hard for [teachers without food allergy (experience)] is that you don’t have the support from someone who knows cause the parent isn’t always *sighs* available, and I think that when you have involved parents, it’s a little bit different. (T19) So much of it is built on relationships, not just with your students. It’s with your community, your families, it’s with your co-teachers, your admin. You have to establish those relationships with so many people, for everything to work. (T10)
*Teacher’s roles*	If I can’t see anything that indicates that it’s made in a peanut-free facility, then unfortunately, I wouldn’t let that kid have it. If it was something that I think could be like a potential allergen, then I might send [treat] home with the student. I would have to use my judgement for the situation. (T11) When you see barriers to food access, are you going to fall on the sword of food allergy and say “You can’t have this, or you can’t eat this”, or are you just going to put them in your office and [clean] the heck out of your office to make sure they’re okay and to make sure [student] got [allergenic food] out of their mouths? (T17)
*Child’s evolution toward self-management*	I think if students have allergies, very early on they should be able to know that, and identify that. So if I had to put a number on it, I’d say as early as kindergarten. (T9) In Kindergarten, that’s also particularly, um, a bigger challenge, because we don’t have these kids prior to Kindergarten. So it’s kind of the first year we’re just starting to get to know them and their unique allergies. (T10) The child [with food allergy] was also more capable and more independent than I think the parents realized, or gave the child credit for. (T14)
*Teachers’ food allergy-related experience*	Its one thing to know [feeling like you’re on the outside] on an intellectual level, but it’s another to walk that, and experience that. […] I’ve had a lot of food sensitivities for years, and I now myself have a food allergy. And even with having people around me with […] significant severe anaphylactic allergies, it wasn’t until I experienced it myself for the first time that I think I really, truly understood what [having food allergy] is like, and how difficult that can be. (T14) There were no food allergies in my family or in my immediate family. So that was never part of our experience growing up. (T15)
*Teachers’ food allergy-related attitudes and beliefs*	I feel that it [food allergy] is a little daunting at the beginning of the year […] The beginning of the year is kind of the worst of it. Where it’s like OK, um you know maybe [parents] didn’t see the note, or just following up with parents, making sure that they’re aware [of food restrictions]. (T2) I feel like it’s almost like innuendo, like it’s [food allergy] something that you should know but it’s not said explicitly. It’s implicit. (T16) I recently did math shapes using marshmallows and toothpicks. And I have a kid with an egg allergy. Do marshmallows have eggs? And I’m like talking to some of the other teachers and going through the list of the ingredients. Things I’ve done in the past, involving food, giving [students with food allergy] that different sensory experience, and there’s things I’ve had to modify. And I choose to modify because you could easily say, well, just give the other kids [with allergy] something else. But, I think also having experienced [having food allergy] myself, I want to make sure that I’m being inclusive to the whole class but still trying to find ways to include those experiences. (T10)

Abbreviations: *T* = teacher

Teachers with direct or indirect personal experience had perceived awareness and cautiousness that helped shape their confidence and competence to manage food allergic reactions. None of the teachers received food allergy training during the course of their university education. Food allergy education was frequently introduced to teachers during URIS training. Some teachers even reflected that their perceived confidence and competence related to anaphylactic management may have been caused by never having had to deal with an emergency situation.

When asked how they think they would handle an emergency situation, teachers described how they thought they would rely on school staff and administrators to help manage the other students and provide emergency treatment if the teacher was not able to.

Other students’ behaviour, changing medical diagnoses, families’ socioeconomic status and school’s reliance on meal programs also impacted teachers’ decision making related to food allergy management. To manage an emergency, teachers described asking families for food allergy-related information to guide their decision making in the classroom, especially for teachers who had no food allergy-related experience. As one teacher eloquently stated, *“At the end of the day, your responsibility is to your students, first and foremost.” (T10)* Thus, teachers talked about adapting lesson plans and integrating concepts of “*safety*”, “*inclusivity*” and *“encourage [students] asking questions” (T10)*, to enforce and educate the class about food allergy.

#### Theme 3: “food allergy could be a more prominent conversation” for teachers to “debunk the myths”

This theme describes the lack of standardization of food allergy education for teachers (Table 4). Teachers received one training session in September through the URIS program for food allergy management among other chronic diseases. But, the session did not comprehensively address teachers’ knowledge gaps.

**Table 4:** Qualitative themes, summary statement, codes and supporting quotations related to Theme 3: “Food allergy could be a more prominent conversation” for teachers, staff and administration to “debunk the myths”

**Theme description:** This theme encompasses the lack of consistency and standardization of allergy education for teachers, staff and administration between private schools, public schools, public schools within the same school divisions, and classrooms within same school. This includes the Unified Referral and Intake System (URIS) education and other (if any) training for staff.	
**Codes**	**Supporting Quotes**
*Teachers’ acceptability of current training*	We only do [training] once a year and if you’re not here, you miss it, you get sent the video that they record. […] I feel like that […] people are kind of missing out sometimes. (T7) [Food allergy could be] a more prominent conversation […] at the beginning of the school year when we get our new kids. […] You’re in your in your first two weeks of school and that’s a crazy time for teacher. And you’re staying after school for this [provincial] training. It just seems like they’re not placing the proper emphasis on [training] ‘cause it is very important. So you can go through the whole year and be like, “That was great, I had my training. I was prepared if something happens.” But nothing happens. So maybe that’s why I felt fine. Right, like if something were to happen mid-year, would I still feel comfortable and remember how to use an [auto-injector] type thing? (T20) We had to watch a video in terms of how to inject [EAI], they don’t talk about what the allergies are and how it works. They talk about what to do if you get a reaction. And that’s pretty much the extent to where they go. (T16)
*URIS program*	We have [URIS training] at the beginning of the year. In our division, we have the URIS nurse that comes and speaks to us, but that’s not before we see our kids, it’s usually a few weeks after we see our kids. (T20) I learned what to do if somebody was to have a reaction. I didn’t really learn about what causes [reactions] or what does it mean to have a peanut allergy. (T16) COVID has actually changed the way URIS looks this year. So URIS group B looks different this year for standard healthcare plans because they have the URIS nurses on, but they’ve been redeployed but I think they’re gently coming back to the URIS program because uh COVID is stabilizing (17)
*Resource needs*	I’m just wondering if kids you know, from all different grades, from all different classrooms, from all different parents whether they’re [families for whom English is an additional language] or not… Do they have those resources to talk to their kids to make sure they’re being vocal and confident about their food allergy? (T8) If there were more kid-friendly ones or family-friendly ones that I could try and I think that could definitely help me having to maybe micromanage less (T2)

Abbreviations: *COVID* = Coronavirus Disease 2019; *EAI* = epinephrine auto-injector; *T* = teacher; *URIS* = Unified Referral and Intake System

Teachers had split opinions whether the training was acceptable. Some teachers reported feeling like the importance of training was disregarded because training was scheduled at the busiest time of the school year, while some teachers reported not recalling whether anaphylaxis management was taught. Teachers reported they *“haven’t gotten any training or anything like that. It’s just sort of like, someone in passing telling us something.” (T7)* Teachers felt like they were “*in the dark in terms of what [food allergy] is” (T16)* and how to prevent allergic reactions. Conversely, other teachers reported the URIS training provided sufficient information.

Teachers unanimously wanted more education sessions throughout the year. Teachers desired further information on signs and symptoms, severity of disease and tolerance, preventative practices (i.e., label reading) and emergency treatment. Teachers also wanted information to share with families, including families for whom English is an additional language (EAL), such as affordable allergen-free foods and resources in multiple languages.

#### Theme 4: communication between all parties is essential

Teachers managed food allergy through relationships and effective communication with school staff, families, students, and the URIS nurses (Table 5). However, teachers wanted for more consistent communication methods. Between staff of the same school, teachers reported of varying communication methods to convey food allergy-related messaging. Handouts for families to communicate allergen-specific bans were often teacher-initiated.

**Table 5:** Qualitative themes, summary statement, codes and supporting quotations related to Theme 4: Communication is a multi-way street between all parties

**Theme description:** According to teachers, effective communication relies on many stakeholders including other teachers, administration, other staff, families and students. Accounts of food allergy-related bullying was observed but teachers believed ongoing open conversation about food allergy with all students helped build safe spaces to prevent bullying. Specifically, communication with families who have EAL needs to be focused on as there are identified communication gaps in addressing food allergy-related topics such as foods allowed in the classroom. Teachers identified ideally using infographics, obtaining translator resources in multi-media sources. Communication gaps between teachers and other staff also put children with food allergy at risk of reaction, especially in situations when the teachers are not directly supervising their students (i.e., lunch break, students are moving between classes).	
**Codes**	**Supporting Quotes**
*Internal communication*	There’s red stop signs near the entrances of the classroom just to say that someone in this classroom has a severe food allergy. It is a whole school plan on how we, um, just communicate with each other, give each other reminders about which class has those severe food allergies. (T8) I’m talking to our food coordinator like, “Don’t give it to the other [grade] 1/2 class cause that boy has peanut allergies” and “That grade 5 class there’s a boy with peanut allergy”. “So can he eat the crackers, can I feed him cheese?” Like I get questions like that, [and teacher says] “Yeah, come check with me if you need but like most things are fine except like this granola, or like sometimes cookies.” So those are the questions that come to me more, is like, what can I feed this child? Well, most things probably don’t have fish but I’m really glad you’re asking. (T19)
*External communication*	It’s [food allergy communication] usually a part of the package that I send home at the beginning [of the school year]. I also verbalize it [to parents] making sure that they do understand that [the school’ is a no peanut kinda situation um, or no whatsoever. It’s clearly outline. (T16) [Food allergy] can be really hard to communicate with parents who come from communities where [food allergy] just doesn’t exist, or they just don’t know the English word for it right. (T7) I received communication back once where the parent was very upset and said [the child’s sandwich] wasn’t peanut butter. It was a [nut spread alternative]. My response was, I’m not with the kids at lunchtime. I’m sorry that happened. I will communicate to [lunch supervisor] that it’s [nut spread alternative]. (T12)
*[Food allergy-related bullying] has never been brought to my attention but “you gotta shut down that real quick”*	You gotta shut that down real quick. And then you go to pull the kids to the side afterwards, who were [bullying], sit them down and explain to them and teach them about […] why it’s not a joke and explain to them why it’s not funny, and [food allergy] is actually very serious. (T13) I seen the little micro-aggressions of kids saying, “Why don’t you just go eat a peanut butter sandwich?” “Why don’t you just go eat a peanut?” […] I haven’t seen a situation where a child has intentionally put in an allergen in another child’s lunch, or in their food or wherever they’re going to be eating or drinking. But, how I’ve handled that in the past is I have held the child back who was saying those things and I had a conversation with them to say that, “This is very serious, this is something that I’ll be talking to the principal about, and this is something I’ll be talking to your family about, because my job is to keep you safe at school, and my job is also to keep your peers safe. And if you’re saying these things and it eventually escalates to acting on it, this could result in that other person being badly hurt.” (T17) I’ve seen less so bullying at that stage but more where so the kids assume what that kid can and can’t have or can and cannot do. And depending on the personality of the child with the allergies, they might sort of go along with that. Or another child might say, “Oh they can’t do that because they have allergies” or “They can’t eat this”. (T14)

Abbreviations: *T* = teacher

The URIS program and schools liaise to create a list of students with chronic disease, to identify which students have a medical condition, and provide the schools with a standardised copy of the student’s healthcare plan. Some teachers did not recollect, or talked about, having these resources, while the teachers who talked about it described these resources as inaccessible should an emergency outside of their classrooms. *“Some students are in music, or in gym, or wherever they might be, and […] other teachers may not be so familiar with that student’s healthcare plan.” (T5)*.

Teachers were the main communication liaison between school staff and families. When there is uncertainty regarding safety of foods brought to school (i.e., treats for class parties, foods brought contained a banned allergenic food), teachers contacted families. At lunch time, most teachers are also on break. Lunch supervisors and/or EAs supervise students, which occasionally resulted in confusion and miscommunication between staff, families and teachers. Specific to EAL families, teachers described food allergy can be “*really hard to communicate with parents who come from communities where [food allergy] just doesn’t exist.” (T7)*.

Food allergy-related communication to students also differed amongst teachers. Teachers speculated they would use different approaches to educating and providing discipline, depending on their students’ needs, if bullying were witnessed. One teacher explicitly recalled having witnessed food allergy-related bullying. *“I [have] seen the little micro-aggressions of kids saying, “Why don’t you just go eat a peanut butter sandwich?” [to the child with food allergy] […] I haven’t seen a situation where a child has intentionally put in an allergen in another child’s lunch.” (T17)*.

## Discussion

To our knowledge, this is the first study to qualitatively explore elementary school teachers’ perceptions on food allergy management. Teachers likely have some degree of health literacy at baseline, and are primarily responsible for supervising and caring for children with food allergy, among other health conditions, for most of their waking hours at school. Yet, teachers have minimal food allergy policies and training provided. In our study, we identified four themes that underscored teachers’ perceptions and experiences managing food allergy.

Themes 1 and 2 highlighted teachers’ experiences navigating the inconsistent food allergy-related policies among Winnipeg schools, and teachers’ perceived lack of knowledge and confidence. Most teachers did not have experience managing anaphylaxis however teachers speculated different ways to manage it if an emergency was encountered. Themes 3 focused on the lack of, and the need for, standardized food allergy education. Teachers had varied experiences that may be attributed to personal experiences, and URIS-provided education on anaphylaxis management, among other sources. These themes collectively highlight the juxtaposition between structured approaches to students (i.e. discipline, behaviour management, assessment) and incoherent food allergy-related education and policies.

Unfortunately, inconsistent food allergy management practices and related policies have been previously reported in the literature [13, 14]. In a recent scoping review from our group, based on studies from Canada, the United States, Australia, and Europe, teachers had variable baseline knowledge, and confidence and self-efficacy, and qualitatively discussed feeling insecure about managing an emergency [13]. Classroom management of food allergies also varied widely, even within individual studies. For example, Eldredge et al. reported that an estimated 25% of participating schools in Wisconsin, USA had no guidelines, whereas others had detailed policies and required individual action plans [25]. Teachers reported having poor knowledge on food allergy management but acknowledged its importance and desire to learn more about preventative and emergency management practices [13]. Teachers in our study described similar needs. At baseline, teachers demonstrated some degree of knowledge, acquired from previous (e.g. URIS) training, which is available to all Winnipeg teachers. This training is mandatory, however, it is brief and is embedded within training for other chronic conditions that require management in school. Additionally, if a teacher is absent from work on the training day, they would have to actively request training or learn from their colleagues who attended. Teachers reported learning how to administer an EAI but teachers also demonstrated inconsistent knowledge of available resources (i.e. URIS program, list of children with chronic disease, location of EAI) in their schools. Interestingly, teachers who had more experience (i.e., lived experience), appeared to have more knowledge in preventing anaphylaxis, and managing food allergy.

Theme 4 emphasized the importance of effective communication amongst all parties. Teachers described relying on families for food allergy-related information and contacting families if questions arise related to food brought into the classroom. Teachers also created their own resources and handouts to communicate food allergy-related information.

Bullying is an aggressive, repeated behaviour that may be verbal, physical, cyber and/or social in nature [26]. In our study, some teachers described witnessing such behaviours, although they did not label is as bullying per se. This is concerning, as teachers may witness food allergy-related bullying, but may not perceive it as such. Of note, school-based, food allergy-related bullying has been reported elsewhere [27, 28]. Yet, a recent quantitative study of Winnipeg-based teachers further supported that teachers believe that food allergy educational topics are of varying importance. While approximately 55% of teachers noted that food allergy was important overall, a majority reported that prevention (e.g. preventing cross-contact between foods), management (recognition of reactions, use of an EAI) and awareness (seriousness of food allergy) were important, while only about half reported that food allergy-related bullying should be discussed [29].

Further, teachers identified areas wherein communication can be improved. Teachers in our study have reported miscommunication with families, and other school staff. This is concerning as miscommunication may put students with food allergy at risk of an allergic reaction. This call for open communication echoes calls from a previous qualitative study, involving parents of children with food allergy, in two Canadian provinces [30]. In one province, namely Ontario, parental advocacy was reduced subsequent to the implementation of a province-wide act requires safeguards to be in place to support students at risk of anaphylaxis. In contrast, the neighbouring province of Quebec lacked legislation regarding food allergy management in schools, and which resulted in parental perceptions of feeling “threatened by the variability and inconsistency of school policies” (p.238). Yet, the need for open communication persisted across both provinces when “dealing with uninformed people” (p. 237) [30]. Thus, policies alone insufficiently contribute to consistent policy. Ongoing training of food allergy and anaphylaxis management must also remain, even subsequent to policy or legislation to protect children at risk of anaphylaxis. Of note, anaphylaxis mismanagement due to the lack of standardized communication practices and/or a lack of available EAI additionally increases the risk of fatal anaphylaxis [1], thereby reinforcing the need to develop consistent, and uniformly applied policies coupled with ongoing education.

Our study involved teachers based in Winnipeg, which is the capital city of the Province of Manitoba. Unlike the Provinces of Ontario [31] and Alberta, [32] which have legislation enacted to protect students at risk of anaphylaxis, no such legislation exists in Manitoba. Moreover, food allergy-related policies differ across intraprovincial jurisdictions. For example, one school division has a policy mandating stock epinephrine autoinjectors, [33] this is not applicable to all Manitoba schools. Of note, all Manitoba-based teachers and school staff are required to complete URIS training, the content of which was developed in consultation with health professionals with expertise in anaphylaxis and community health [34]. While there are no publicly-available intended learning outcomes for the URIS program, the anaphylaxis manual details food allergy management practices, including consideration to age-appropriate practices, as well as anaphylaxis recognition and emergency treatment [34].

Our study results echoed the themes identified in Hinton & Kirk (2015)’s narrative review of teachers’ perspectives teaching children with chronic disease. Although this review focused on students with asthma, epilepsy and diabetes, teachers in this study wanted and needed more education, training and resources to increase their confidence to teach, care for and manage their students with chronic disease. Expectedly, increased communication and educational programs were deemed beneficial for teachers [35], In our study, all teachers spoke about benefitting from more frequent education and emergency management training. This suggests that teachers, regardless of whether they had previous experience of food allergy or not, valued shared knowledge and shared responsibility amongst families, students and all school staff.

Teachers also collectively voiced their recognition and want for standardized food allergy education. Food allergy education would be beneficial for all paid adults in the school, who may witness a food allergy emergency in the school, including student teachers, substitute teachers, lunch supervisors and all support staff. Further, providing standardized food allergy resources (i.e. infographics, emergency plan) in every classroom may facilitate better decision making and increase confidence should an emergency occur.

Like all qualitative studies, our study findings are not generalizable but may be transferable to similar populations. Our participants taught mostly younger grades (K-Grade 3), therefore, these findings may not be transferable to teachers who have older students. While a few teachers in our study described witnessing bullying-like actions, these actions were not widely described, possibly as these teachers taught younger children. In contrast, self-reported food allergy-related bullying amongst older school children has been reported as being common, with as many as one-in-three youth reporting such bullying [26]. We also note that teachers who participated in our study were likely more motivated to speak and learn about food allergy, while some teachers may perceive food allergy management as being beyond their current scope or bandwidth.

A strength of this study is reporting an in-depth analysis of 16 teachers’ perceptions and experiences managing food allergy in their classrooms and schools. Layers of lived experiences and pedagogical principles ultimately shape the way teachers experience, perceive and therefore create the truth (25). Through these interviews, we identified ways in which food allergy management can be improved by way of more education and training related to prevention and treatment of food allergic reactions, better and ongoing communication between and amongst relevant parties and standardization of policies related to food allergy, including recommendations on how teachers should manage unintended consequences or situations related to those policies. Teachers’ interest to participate in these interviews in a time when schools were subjected to many COVID-19-related changes [20] was also a study strength. In addition, these interviews were conducted during a time when birthday parties and field trips, were not possible due to COVID. Mentioning these events during interviews, in relation to food allergy, spoke to how much teachers thought, and perhaps worried, about food allergy management though special occasions were not as relevant during the pandemic.

## Conclusion

Many factors influence teachers’ decision decision-making in the school and in the classroom to reduce the risk of allergic reactions, food allergy-related bullying and creating a safe, inclusive learning space for all students. Teachers manage food allergy in their classrooms by making decisions to prevent food allergic reactions, as informed by their knowledge and lived experience, guided by the current policies that surround their schools. At the same time, teachers consider the individualized needs of their students and rely on families for support. Teachers acknowledged their variable knowledge and experiences, but unanimously wanted more training and resources to better improve their food allergy education and anaphylaxis management skills.

## Electronic supplementary material

Below is the link to the electronic supplementary material.

Supplementary Material 1: Semi-structured interview guide

## Data Availability

Written requests for anonymous data will be considered by the authors.

## Abbreviations

COVID-19: Coronavirus Disease or Novel Coronavirus (2019-nCoV)
EA: Educational assistant
EAI: Epinephrine auto-injector
EAL: English as an additional language
EAP: Emergency Anaphylaxis Plan
K: Kindergarten
T: Teacher
URIS: Unified Referral and Intake System
