# Supplementary material for: Winnipeg-based elementary school teachers’ perspectives on food allergy management: a qualitative analysis
Source: Allergy Asthma Clin Immunol. 2023 Jul 14;19:62. doi: 10.1186/s13223-023-00798-z (PMC10347816; doi:10.1186/s13223-023-00798-z)
Supplement: Supplementary file 1 — Supplementary Material 1: Semi-structured interview guide [file 13223_2023_798_MOESM1_ESM.docx]

**eTable 1**

**Semi-structured interview guide**

1. Introductory questions

- Would you please tell us a bit about your role and background?
- Before becoming a teacher, how much did you know about food allergy?
- Since becoming a teacher, what are your experiences with food allergy?
  - Where did you learn about food allergy?

2. Content questions

- How does your school manage food allergy?
- Please describe your confidence in managing food allergy in the classroom.
- What kinds of supports, training and/or resources do you currently have to manage a severe allergic reaction in your classroom?
- What additional kinds of supports, training and/or resources would you need to manage a severe allergic reaction in your classroom?
- If you ever had an experience working or managing a child with food allergy, please discuss.
- Research tells us that children with food allergy are often bullied or teased because of their food allergy. If you’ve ever seen this, please share your experience and ability/ comfort in being able to handle the situation.
- How do you, as a teacher, feel about being responsible for a food allergic child?
- The COVID-19 pandemic has changed every aspect of our lives. As teachers you have been responsible for your students’ well-beings in ways, we did not imagine two years ago. Please describe any differences in how food allergy is managed in your classroom/ school during the pandemic to before the pandemic.

3. Closing questions

- Of all the topics we discussed today, what is the most important part for you?
- Now, I will provide a brief summary about what we talked about. Please correct me if anything is incorrect. (Provide summary)
  - How was that?
  - Did I capture what you said, or did I make some mistakes? (Correct if necessary)
- We've talked a lot today about working with a child with food allergy. Is there anything that I should have asked, but did not?
  - Would you like to add anything else?
